# The Bisdioxopiperazine ICRF-193 Attenuates LPS-induced IL-1β Secretion by Macrophages

**DOI:** 10.1007/s10753-023-01895-2

**Published:** 2023-09-01

**Authors:** Ashleigh Brindle, Callum Bainbridge, Muganti R. Kumar, Stephen Todryk, Kay Padget

**Affiliations:** Faculty of Health and Life Sciences, Northumbria University at Newcastle, Newcastle Upon Tyne, NE1 8ST UK

**Keywords:** Macrophages, Topoisomerase II, IL-1β, ICRF-193

## Abstract

Inhibiting pathological secretion of Interleukin-1β has shown beneficial effects in disease models and in the clinic and thus there is interest in finding inhibitors that can reduce its release from macrophages in response to their activation by foreign pathogens. We used an *in vitro* human macrophage model to investigate whether ICRF-193, a Topoisomerase II inhibitor could modulate *IL1B* mRNA expression and IL-1β secretion. These macrophage-like cells readily secrete IL-1β in response to Lipopolysaccharide (LPS). Upon exposure to a non-toxic dose of ICRF-193, IL-1β secretion was diminished by ~ 40%; however, level of transcription of *IL1B* was unaffected. We show that there was no Topoisomerase 2B (TOP2B) binding to several IL1B gene sites, which may explain why ICRF-193 does not alter IL1B mRNA levels. Hence, we show for the first time that ICRF-193 can reduce IL-1β secretion. Its low cost and the development of water-soluble prodrugs of ICRF-193 warrants its further investigation in the modulation of pathological secretion of this cytokine for the treatment of inflammatory disorders. (165 words).

## INTRODUCTION

IL-1β is a pro-inflammatory cytokine with a wide range of biological effects and is produced and secreted by several cell types such as macrophages, B lymphocytes, natural killer cells, smooth muscle cells and fibroblasts [1, 2]. Although IL-1β is essential for host-responses to infection and injury, it also exacerbates damage during acute tissue injury and in chronic inflammatory diseases such as cardiovascular disease, osteoarthritis, rheumatoid arthritis, inflammatory bowel disease, type 2 diabetes, multiple sclerosis, and Alzheimer's disease [3, 4]. Hence, therapeutic strategies that suppress the release of IL-1β offer a strategy to regulate inflammation to reduce organ damage and fibrosis [5–7].

Recently, 24 h treatments with low dose Epirubicin (0.17 μM), an anthracycline poison of topoisomerase II (TOP2), has been shown to reduce inflammatory release of IL-1β by macrophages by down-regulating the NLRP3 inflammasome components and reducing the release of cleaved caspase-1. Suppression of transcription of LPS dependent genes with concomitant reduced acetylation of histone 3 lysine 9 (H3K9ac) was also observed [8]. Previously, Epirubicin has been shown to interact with histone H3 and inhibit acetylation of K9/K14 resulting in more compact and transcriptionally repressed chromatin [9]. In addition, anthracyclines have been shown to evict histones from particular regions in the genome, causing epigenomic and transcriptional alterations and attenuated double-strand break (DSB) repair [10–13]. Epirubicin has also been shown to bind to G-C sequences with higher affinity and can inhibit the formation of the transcription pre-initiation complex [9]. Thus, epirubicin can modulate chromatin structure and function.

Epirubicin is also a TOP2 poison. TOP2 enzymes can relieve DNA torsional stress in chromosomes caused by the unwinding of double stranded DNA during normal cellular processes such as replication, transcription, recombination and repair [14–16]. TOP2 achieves this by creating a transient double-strand break in the DNA duplex through which another duplex is passed before the break is resealed [17]. Epirubicin prevents TOP2-mediated DNA strand religation and results in a DSB associated with the enzyme [18, 19].

Mammalian cells encode two isoforms of *TOP2; TOP2A* and *TOP2B* whose genes have been mapped to q12-21 on chromosome 17 and p24 on chromosome 3, respectively [20]. A study using Nalm-6WT, Nalm-6 *TOP2A* ± and Nalm-6 *TOP2B-/-* showed that epirubicin mediates its toxicity mainly through TOP2A [21] and this was supported by a later study using the TARDIS assay which showed very few TOP2B epirubicin stabilised cleavage complexes in comparison to TOP2A using 10 μM epirubicin [22].

TOP2 isoforms are structurally similar, possessing 68% amino acid sequence identity, with 77% of that being conserved in the N-terminal domain [23]. They also appear to be biochemically similar, sharing the same catalytic cycle; however they play different roles in the cell. For example, TOP2A plays an indispensable role in genome replication and mitotic segregation by relieving torsional stress and preventing intertwining of daughter DNA molecules. TOP2 enzymes, particularly TOP2B, also participate in facilitating gene transcription [24–28]. Indeed, inhibition of TOP2B results in ~ 30% of the genes involved in neuronal development to be down-regulated [25] and this is supported by the non-viable TOP2B knockout mouse model [29], which demonstrated abnormal neuronal development. It has also been reported that TOP2B not only preferentially bound to regions containing high H3K4 methylation but also promoters that actively recruit RNA polymerase, both of which are features of transcriptionally active chromatin [30].

Given the role of TOP2 it is conceivable that the attenuation of IL-1β secretion and expression, as seen with epirubicin, could also be mediated in part by the modulation of TOP2 activity. For example, it has been shown that a 30-min exposure to 10 μM epirubicin could inhibit the ability of purified TOP2A and TOP2B from decatenating kinetoplast DNA *in vitro* [22]. To further understand if this is the case we used ICRF-193, a TOP2 inhibitor, to test for mitigation of IL-1β secretion and expression. ICRF-193 is a Bisdioxopiperazine and is non-DNA-intercalating, unlike epirubicin. The drug binds to the interface between TOP2 subunits formed when ATP or ADP is bound, thereby inducing the formation of a closed clamped intermediate of TOP2 on the DNA [31, 32]. ICRF-193 targets both isoforms of TOP2 with a preference for TOP2B [33]. In contrast to TOP2 poisons such as epirubicin, Bisdioxopiperazines have limited anti-cancer activity and only a prolonged period of exposure results in DNA break-related and DNA break-unrelated cellular damage [34, 35]. Instead they are primarily used to attenuate anthracycline cardiotoxicity in the clinic [36]. However as they are the most specific inhibitors of TOP2 that are not TOP2 poisons, they are an essential implement for studying the effect of inhibiting TOP2 [37].

The effect of ICRF-193 on IL-1β secretion and expression was investigated using the continuous human cell line U937, differentiated into a macrophage-like cell type using PMA, a synthetic phorbol ester [38–40]. Although the use of Monocyte-derived Macrophages help overcome the innate difficulties working with primary macrophages such as invasive harvesting methods, heterogeneity and lack of longevity in culture [41–43], it should be noted that the choice of isolation method and differentiating agent can significantly impact cellular phenotype and thus comparisons must be made with care [44]. Nevertheless, we show that these macrophage-like cells readily secrete IL-1β in response to LPS exposure and that upon exposure to a non-toxic dose of ICRF-193, IL-1β secretion was attenuated but transcription of *IL1B* remained unchanged. We show that there was no TOP2B binding to several *IL1B* gene proximal sites, confirming that TOP2B is not involved directly in mediating the transcription of *IL1B* at these sites and hence why ICRF-193 does not alter *IL1B* mRNA levels. We also show that cells in this model system have reduced levels of *TOP2A* and *TOP2B* mRNA compared to their non-differentiated counterparts; however, only TOPA protein levels showed a significant decrease. *Ex vivo* differentiated monocytes derived from peripheral blood also generally displayed this reduction in *TOP2A* and *TOP2B* mRNA levels. Hence, ICRF-193 merits further investigation as a promising drug candidate for controlling IL-1β secretion in inflammatory disorders.

## MATERIALS AND METHODS

### Cell Culture

The U937 cell line was derived from a 37-year-old Caucasian male suffering from histiocytic lymphoma and is characterised as being monocytic. Cells were maintained as a suspension culture in RPMI 1640 medium supplemented with 10% foetal calf serum (FCS), penicillin (50 U/ml), streptomycin (50 µg/ml) and glutamine (2 mM). Cells and were grown at concentrations between 1 × 10^5^/ml and 1 × 10^6^/mL and were free of mycoplasma contamination. Cells were cultured at 37 °C, 5% CO_2_ in a humidified atmosphere. Cell culture reagents were obtained from GIBCO, Life Technologies.

### Peripheral Blood Mononuclear Cell (PBMC) Isolation

A qualified phlebotomist obtained 50 ml of venous blood from consenting healthy volunteers. Ethical approval was obtained from Northumbria University (project reference: SUB86_AJ_0511). Whole blood was added to a Leucosep tube (Greiner) and centrifuged for 12 min at 700 × g with no deceleration. The buffy coat layer was removed and mixed with 50 ml of FCS free-RPMI-1640 and centrifuged at 600 × g for 10 min with maximum deceleration. The supernatant was then discarded, and the remaining cells were re-suspended in 2 ml of FCS free-RPMI-1640.

### CD14 Selection of PBMCs

1 × 10^7^ cells were re-suspended in 50 μl of selection buffer (2 mM EDTA/PBS pH 7.2, 0.5% FCS). 20 μl of CD14 Microbeads (Miltenyi Biotec) were then added to the suspension of cells, mixed and incubated for 15 min at 4 °C. 1.5 ml of selection buffer was then added to the suspension of cells, this was followed by centrifugation at 1500 rpm for 5 min. The supernatant was removed, and cells were re-suspended in 500 μl of selection buffer. At this time a MACS MS + /RS + column was applied to the MACS separator and washed with 500 μl of selection buffer. The cell sample was then added and flow through was discarded. The column was then washed a further 3 times with 500 μl of selection buffer. The column was then removed from the MACS separator and 1 ml of buffer was used to flush out the CD14 + cells.

### Quantitation of CD11b Surface Antigen Expression using Flow Cytometry

U937 cells were harvested and washed twice with PBS containing 0.1% FCS. Cells were then transferred to a FACs tube (Sarstedt) and centrifuged at 1000 rpm for 5 min. PBS was removed and cells were re-suspended in 20 μl of fresh PBS containing 0.1% FCS. 2 μl of Anti-CD11b –APC conjugated antibody (eBioscience 17–0118-41) was then added. Cells were gently mixed and allowed to incubate for 30 min in the dark. Samples were then washed twice with 1 ml PBS and re-suspended in 300 μl PBS. Flow cytometry was then performed.

### Cell Cycle Analysis using Flow Cytometry

U937 were harvested and washed twice with PBS. Cells were then re-suspended in 200 μl PBS and added dropwise to 2 ml of ice cold 70% ethanol in PBS. Cells were fixed for 30 min at 4 °C. The fixative was then removed, and the cells were resuspended in 1 ml of PBS containing 0.2 μg/ml RNase A and 10 μg/μl propidium iodide. Cells were then incubated for 30 min at 37 °C. Flow cytometry was then performed.

### XTT Assay

50 μl of XTT (1 mg/ml in RPMI-1640) and PMS (25 μM in PBS) reagent was added to 200 μl of cells and incubated at 37 °C, 5% CO_2_ in a humidified atmosphere for 4 h. The endpoint absorbance was then read at 450 nm and 630 nm on a spectrophotometer (Synergy, BioTek). Percentage cell viability was determined by the following calculation:

$$\left(Specific\;absorbance\;of\;test\;sample\right)\div\left(Specific\;absorbance\;of\;control\;sample\right)\times100$$

### Trypan Blue Exclusion

Light microscopic quantitation of cell viability was carried out using a previously published method [45].

### ELISAs

Quantification of IL-1β released into the supernatant was performed using the Ready-Set-Go ELISA kit to human Il-1β (eBioscience). A 96 well ELISA plate was coated overnight at 4 °C with an Il-1β capture antibody. The wells were then aspirated and washed 5 times (PBS, 0.05% Tween20.) Standard samples and experimental samples were then added to the plate in triplicate and the plate was then incubated for 2 h at room temperature. The wells were then aspirated and washed 3 times as before. IL-1β detection antibody was added to the plate and incubated for 1 h at room temperature. Wells were again aspirated and washed 5 times as before. The avidin–HRP enzyme was then added to the plate and incubated for 30 min at room temperature before the wells were aspirated and washed 7 times as before. 100 μl of substrate solution was then added to each well and the plate was left to incubate for a further 15 min. 50 μl of 2 M H_2_SO_4_ was then added to each well to stop the reaction. The plate was then read at 450 nM and 570 nM on a spectrophotometer (Synergy, BioTek).

### Western Blotting

Cells were collected at 72 h following treatments and whole cell extracts were prepared [46]. Equal protein loads were applied to each lane and proteins separated using SDS-PAGE. TOP2A was detected with rabbit anti-TOP2A IgG (Sigma AV04007), TOP2B was detected using Mouse anti-TOP2B IgG (BD Bioscience 611,493), GAPDH was detected with rabbit anti-GAPDH IgG (Abcam ab9485) and Actin was detected using mouse anti-actin IgG (Thermo Scientific AC15). Pre-stained molecular weight markers were purchased from Biorad, UK (#1,610,374).

### **Quantitative Real-time PCR of *TOP2A* and *TOP2B* mRNA**

RNA extraction was performed using the High Pure RNA Isolation kit (Roche). Synthesis of cDNA from RNA was performed using the Precision nanoscript reverse transcription kit (PrimerDesign). qPCR was performed using the C1000 Thermal Cycler BIO-RAD CFX96 Real time system using the following Taqman Primer Design hydrolysis probes:

TOP2A: Forward: TGGATTTGGATTCAGATGAAGATTT.

Reverse: CTAAGTTTTGGGGAAGTTTTGGT.

TOP2B: Forward: ATATGTCTCTGTGGTCTCTTACTAAA.

Reverse: GCCGCTAAATCCTCTTTCCAA.

Analysis was performed using the comparative ΔΔCt method [47] to determine relative fold expression between stimulations. 18S RNA was used as the reference gene [48].

### Chromatin Immunoprecipitation (ChIP)

ChIP was performed using the EZ-Magna ChIP A, 17–408 kit (Millipore) and performed according to the manufacturer’s protocol. Briefly, differentiated U937 cells were exposed to LPS (Sigma-Aldrich) for 72 h. Treated and untreated control cells were then fixed using formaldehyde (1%, Sigma-Aldrich) and then neutralised with glycine. Cells were then washed twice in PBS containing protease inhibitors before lysis. Chromatin was fragmented to between 500 – 1000 bp using sonication (14 × 15 s cycles). 5% of the sample was removed as ‘input’. Anti-TOP2B (30,400), or anti-GFP (Santa Cruz Biotechnology, sc-8334) or anti-AcH3 (Millipore, 06-599B) were added to protein A magnetic beads (Invitrogen, 100.02D) and incubated for 30 min at room temperature. Chromatin samples were then added to the beads and incubated for 18 h at 4 °C. Protein A Magnetic beads were then separated from the rest of the sample using a magnetic separator. Immunoprecipitated protein-DNA complexes were then washed sequentially in a series of wash buffers provided in the Magna EZ ChIP kit as follows; Low salt Immune Complex Wash buffer, High Salt Immune Complex Wash buffer, LiCl Immune Complex Wash buffer and TE Buffer, allowing 5 min for incubation for each wash. Crosslinks in all samples including the ‘input’ were reversed by incubation in elution buffer with Proteinase K for 3 h at 62 °C with rotation. The samples were then heated to 95 °C for 10 min. DNA was then purified using a spin column (Millipore). qPCR was performed using iQ SYBR green supermix (Bio-Rad, 10,003,253), primers are shown in Table 1. Results were quantified as % input with GFP and AcH3% input values being the negative and positive controls, respectively.

**Table 1:** Primer Pairs for *ILIB* ChIP analysis

Sequence ID	Forward primer sequence	Reverse primer sequence
*IL1B -1*	TTACTTGGCACCCTGTTTGC	ATTACAGGTCAGTGGAGACGC
*IL1B -2*	AGCTATGATGGGTTCACCGC	AAACAAGAGTGCTGGAGCGA
*IL1B -3*	TCTGTTCCCTTTCTGCCAGC	AGCAGAAACATGCCCGTCT
*IL1B* control	TTACTTGGCACCCTGTTTGC	ATTACAGGTCAGTGGAGACGC

## Results

**Fig. 1:**
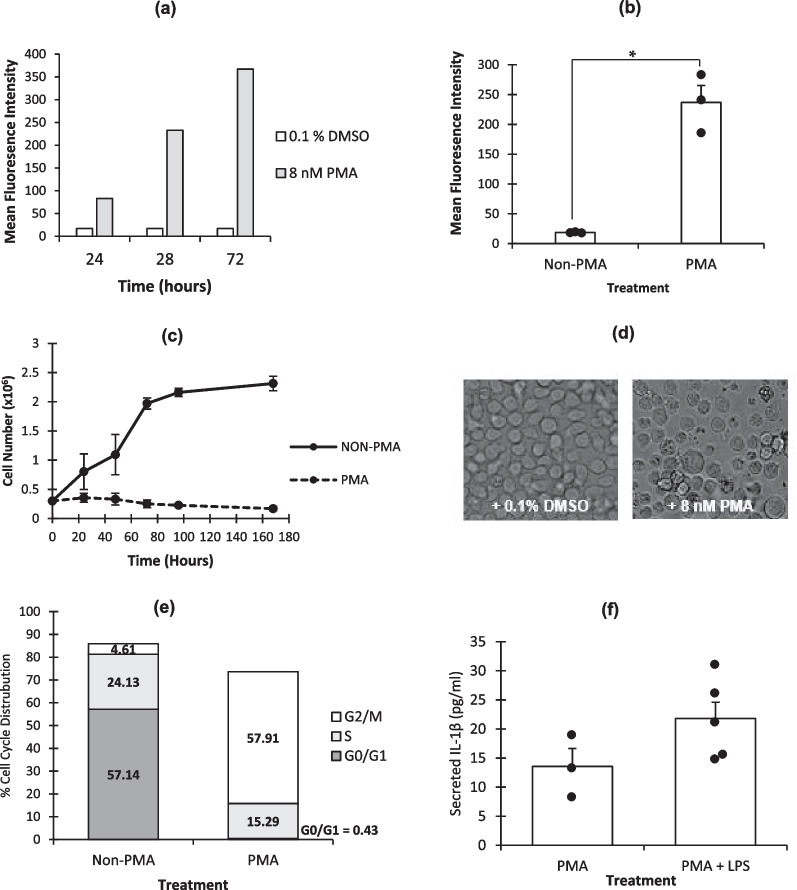
**CD11b Macrophage Marker Expression, Cell Proliferation and IL-1β Secretion in Response to PMA/LPS**. **a **Cells were exposed to 8 nM PMA or PMA vehicle control (0.1% DMSO) for 24, 48 and 72 h then harvested and labelled with anti-human CD11b-APC conjugated antibody and flow cytometry was performed. Results are presented as the mean fluorescence intensity of 1 × 10^5^ captured cells. **b** The means ± Standard Error (SE) of three independent biological replicates of cells exposed to 8 nM PMA for 72 h and 0.1% DMSO. * = p < 0.01 (2-tailed, unpaired t-test). **c** Proliferation of cells exposed to 8 nM PMA or 0.1% DMSO was measured after 24, 48, 73, 96, 168, and 196 h using the trypan blue exclusion method and means ± SE of three independent biological replicates are shown. **d** Visual analysis of PMA versus DMSO treated U937 cells for 72 h. **e** Cell cycle analysis of DNA content using propidium iodide staining on non-PMA and PMA treated cells for 72 h. Percentage of 10,000 captured cells in each phase was then calculated. **f** Cells were seeded at a density of 3 × 10^5^/ml with 8 nM PMA alone or 8 nM PMA and 1 ng/ml LPS for 72 h before the supernatant was harvested. An ELISA to human IL-1β was then performed. The means of three independent experiments are shown ± SE.

As all macrophages, including classically activated macrophages (M1) and alternative activated macrophages (M2), are known to express the cell surface antigen, CD11b [49, 50], this was used as a marker of differentiation of the U937 cells into a macrophage-like state. To determine the optimal PMA exposure time to induce the cell type to the terminal monocytic differentiated state, CD11b levels were monitored as levels steadily increase through the differentiation stages [51]. Results showed that CD11b expression was highest at 72 h (Fig. 1a) and showed significantly upregulated CD11b expression (Fig. 1b).

Increased expression of CD11b was also accompanied by a reduction in cell proliferation, as measured by trypan blue exclusion (Fig. 1c), and an increase in the visual granularity and size of the cells (Fig. 1d). In addition, an increase in the proportion of cells in G2M consistent with the macrophage-like phenotype was also seen in these cells (Fig. 1e) [52]. Cells also became adherent. Taken together, these results confirmed a differentiated-like phenotype. Cells exposed to PMA for 72 h also secreted bioactive Il-1β as measured by ELISA (Fig. 1f). When co-incubated with LPS, which polarizes the cells towards the M1 phenotype, more Il-1β secretion is noted in line with previous studies [53, 54]. However levels of secreted IL-1β are lower than many published studies which use shorter pulses of higher dose LPS ranging from 100 – 1000 ng/ml [55, 56]. A low dose of LPS was selected as subsequent experiments using co-treatment with LPS and ICRF-193 were chronic 72 h exposures and we wished to avoid a change of cellular phenotype from pro- to anti-inflammatory [57, 58].

**Fig. 2:**
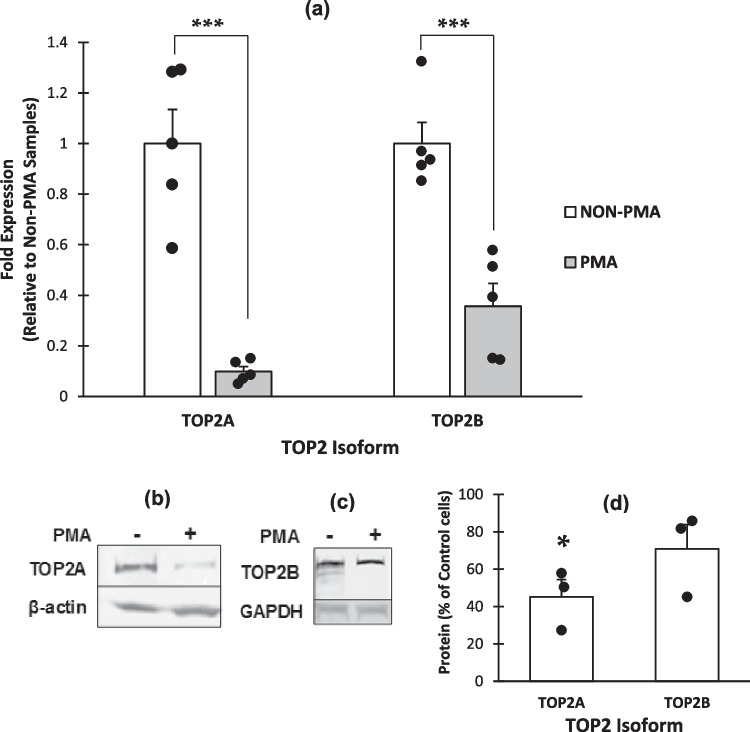
**Comparing the relative levels of *TOP2A* and *TOP2B* mRNA and protein in Non-PMA and PMA treated U937 cells.** Cells were seeded at a density of 3 × 10^5^ cells/ml with 8 nM PMA or 0.1% DMSO (non-PMA) and allowed to incubate for 72 h before harvesting. RNA was then extracted and cDNA synthesised. qPCR was then performed using Taqman hydrolysis probes to *TOP2A* and *TOP2B ***a**. Results were normalised to 18S following which the ΔΔCt method was used to calculate fold expression. Results shown are the mean of five independent experiments ± SE.*** = p < 0.001 (2-tailed, unpaired t-test). Whole cell protein was also extracted and equal protein loads were separated by SDS-PAGE, transferred to nitrocellulose before being probed with antibodies to TOP2A **b** and TOP2B **c**. Representative images of three independent experiments are shown. **d **Quantification of relative levels of TOP2A and TOP2B protein was performed using the densitometry software, Syngene genetools, data is presented as amount of protein in PMA treated cells as a percentage of that in non-PMA treated cells. * = p < 0.05 (2-tailed, unpaired t-test).

During cellular differentiation, the level of both TOP2 isoforms are known to change [59–63], therefore to ascertain the levels of the two isoforms in U937 cells following treatment with PMA alone, TOP2A and TOP2B protein and mRNA levels were semi-quantified (Fig. 2).

**Fig. 3:**
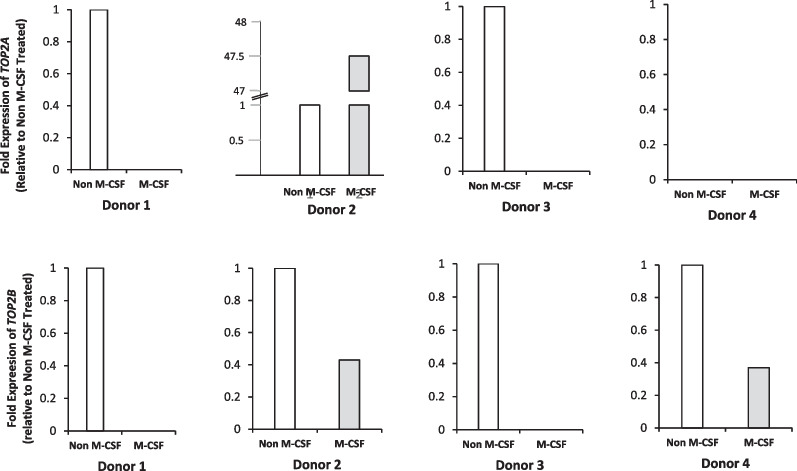
**Fold expression of *TOP2A* and *TOP2B* in CD14 + monocytes from Peripheral Blood ± M-CSF.** CD14 + monocytes derived from peripheral blood from 4 donors were seeded at 1 × 10^6^/ml in RPMI-1640 with 50 ng/ml M-CSF or 0.1% DMSO (M-CSF vehicle control). Cells treated with 0.1% DMSO were left to incubate for 24 h before harvesting. M-CSF treated cells were left to incubate for 7 days before harvesting. RNA was extracted and cDNA synthesised. qPCR was performed using Taqman probes to *TOP2A* or *TOP2B* and normalised to 18S and samples analysed in triplicate. Results are shown comparing non-MCSF and MCSF treated CD14 + monocytes from each individual donor.

*TOP2A* and *TOP2B* mRNA levels both decreased significantly upon treatment with PMA for 72 h and was accompanied by a decrease in both proteins; however, only TOPA protein levels showed a significant decrease. A reduction in *TOP2B* mRNA is seldom reported in the literature in differentiated versus non-differentiated cells, with most reporting an upregulation [25, 30, 59]. This reduction may be a cell type specific phenomenon, so we investigated whether this decrease in both *TOP2A* and *TOP2B* mRNA is also seen in normal peripheral blood monocytes when differentiated. To do this, we isolated monocytes from the blood of healthy volunteer donors and differentiated them using M-CSF alone and quantified *TOP2A* and *TOPB* mRNA levels using qPCR (Fig. 3).

TOP2A mRNA could be detected in only half of the non M-CSF samples examined and no TOP2A mRNA was seen in all of the samples cells exposed to M-CSF, except one. In contrast TOP2B mRNA was detected in all of the non M-CSF samples examined. However, the levels of *TOP2B* mRNA in M-CSF treated cells from donors 2 and 4 show a decrease and data sets of donors 1 and 3 demonstrate a lack of amplification of *TOP2B* after treatment with M-CSF, suggesting that levels are so low they are undetectable. These results support data seen in the U937 cells that show *TOP2B* mRNA levels decrease when these cells are differentiated into macrophage-like cells.

**Fig. 4:**
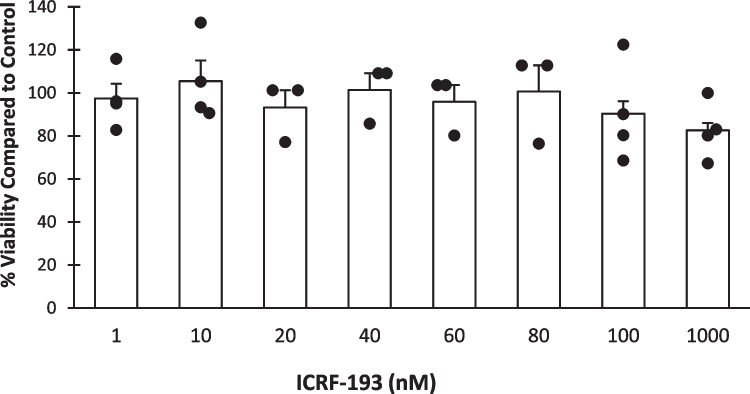
**The cytotoxic effect of ICRF-193 on PMA treated U937 cells**. Cells were seeded at a density of 3 × 10^5^cells/ml with 8 nM PMA and allowed to incubate for 72 h. Media was then replaced with the addition of varying concentrations of ICRF-193. 0.1% DMSO was used as the drug vehicle control. Cells were allowed to incubate for a further 72 h, after which an XTT assay was performed. Results are reported as percentage of 0.1% DMSO vehicle control and are the mean of at least three independent experiments ± standard error. No significant decrease in cell viability was detected at any dose.

To investigate the effect of inhibiting TOP2 on IL-1β secretion and expression levels, cells were co-treated with PMA, LPS and ICRF -193. Cells were exposed to either 1 nM or 150 nM ICRF-193. These doses were confirmed to be non-toxic to the PMA treated cells (Fig. 4).

**Fig. 5:**
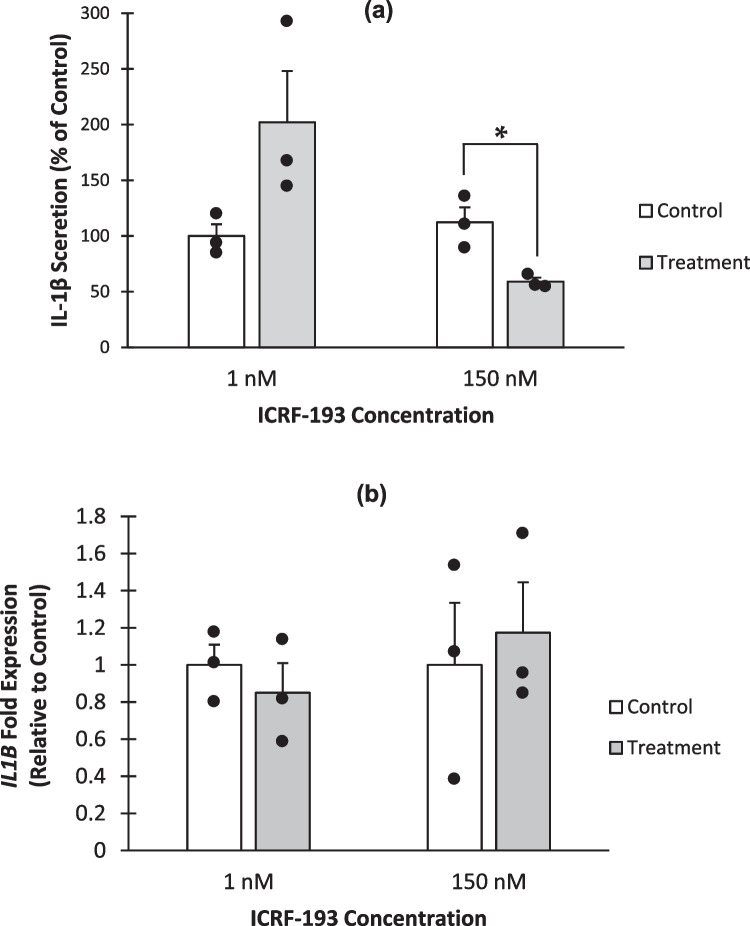
**IL-1β secretion and mRNA levels following co-treatment with LPS and ICRF-193.** U937 cells were seeded at a density of 3 × 10^5^/ml with 1 nM ICRF-193, 150 nM ICRF-193 or 0.1% DMSO. 8 nM PMA and 1 ng/ml LPS were also added. **a** Cells were incubated for 72 h before the supernatant was harvested. An ELISA to human IL-1β was then performed. Data is presented as percentage of the 0.1% DMSO treated control. The means of four independent experiments are shown ± SE. * = p < 0.05 (2-tailed, unpaired t-test). **b** Cells were incubated for 72 h before harvesting. RNA extraction and cDNA synthesis was performed, followed by qPCR using a hydrolysis probe to *IL1B*, using 18S as the reference gene. Fold expression was calculated using the comparative ΔΔ Ct method. Data is presented as percentage of the 0.1% DMSO treated control. The means of three independent experiments are shown ± SE.

U937 cells were co-treated with 1 nM or 150 nM ICRF-193, along with PMA and LPS for 72 h and human IL-1β protein secretion and levels of expression were made using ELISA and qRT-PCR, respectively (Fig. 5).

**Fig. 6:**
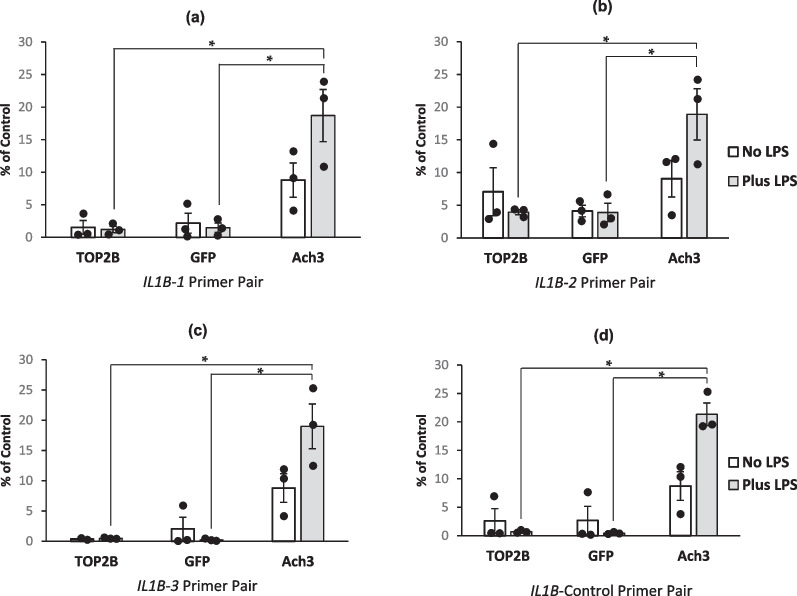
**Determining if TOP2B association increases at sites proximal to the promoter region of the *IL1B* gene when PMA treated cells are stimulated with LPS.** Cells were seeded at a cell density of 3 × 10^5^ cells/ml with 8 nM PMA with and without 1 ng/ml LPS and allowed to incubate for 72 h before being harvested. Cells were fixed and ChIP was performed. Immuno-precipitations used in the ChIP utilised antibodies to TOP2B (target), Green fluorescent protein (negative control) and Acetylated histone 3 (positive control). qPCR was then performed using 4 primer pairs (see materials and methods), *IL1B-1 ***a**, *IL1B-2 ***b**, *IL1B-3 ***c** and *IL1B* control **d**. Results are reported as percentage of input (no IP performed on this sample). The means of three independent experiments are shown ± SE except for the *IL1B-3* primer pair and TOP2B antibody where only 2 replicates are shown * = p < 0.05 (2 tailed, unpaired t test).

IL-1β release was reduced by 41% (p = 0.03, student t-test) by treatment with 150 nM ICRF-193 (Fig. 5a). However, the attenuation of IL-1β protein secretion by ICRF-193 was not mediated at the level of *IL1B* mRNA as levels were unaffected by the treatments, as shown in Fig. 5b. As TOP2B is known to be involved in mediating transcription of some genes [24–28], and as ICRF-193 has a preference for inhibiting this isoform [33] and causing its degradation [64, 65], we wanted to confirm that TOP2B was not involved with transcription of *IL1B*. ChIP-qPCR was therefore utilised to analyse any TOP2B interactions with the *IL1B* promoter (Fig. 6). Three primer pairs used in the analysis were designed based on known DNAse hypersensitivity sites and putative TOP2B cleavage sites within the *IL1B* gene promoter (Fig. 6a-c). *IL1B* control primers (Fig. 6d) were designed to amplify regions which did not contain a DNase hypersensitivity or a putative TOP2B binding site and was located downstream of the promoter region within the gene [30].

Results showed that there was no significant difference in TOP2B association between the non-LPS treated samples and the LPS treated samples when using any of the primer pairs designed to amplify regions proximal to the promoter, thus suggesting there is little or no detectable TOP2B at the region of the *IL1B* gene that the primers amplify. Percentage input of the matching positive controls in the presence of LPS were significantly higher than both that seen with the TOP2B primers or the negative controls (GFP).

## Discussion

We sought to determine if the inhibition of TOP2 activity would decrease IL-1β secretion and expression, and confirmed that ICRF-193, a TOP2 inhibitor, significantly decreased the amount of bioactive IL-1β secreted by cells displaying a macrophage-like phenotype, as measured by ELISA. Cells that had been co-treated with 150 nM ICRF-193, PMA and LPS for 72 h displayed a significant decrease of 41% in IL-1β protein secretion (p = 0.03). However, unlike epirubicin, a TOP2 poison and DNA intercalator, expression of *IL1B* mRNA was unaffected by treatment with this drug. This suggests that TOP2 is not involved in modulating *IL1B* mRNA expression. As TOP2B is the major isoform that is involved in transcriptional regulation [24–28], it was decided to test if TOP2B was associated with the promoter region of *IL1B* in the presence of LPS. ChIP experiments confirmed that TOP2B was not associated with the specific regions tested, suggesting that TOP2B may not be involved in transcription of *IL1B* in response to LPS stimulation in these cells which may explain why these doses of ICRF-193 do not inhibit transcription of *IL1B*. However, ChIP-sequencing would be needed to identify if other TOP2B binding sites occur across the *IL1B* gene to fully explore this. In addition direct interaction between TOP2B and ICRF-193 in this context would require verification and whether its disruption leads to secretion of normal levels of IL-1β.

Although *TOP2A* and *TOP2B* mRNA levels both decreased significantly upon treatment with PMA for 72 h, only TOPA protein levels showed a significant decrease, suggesting that levels of TOP2B protein remain available to inhibition by ICRF-193. In contrast to this present data many studies using both primary and continuous cells have shown that *TOP2B* mRNA and protein level are increased upon differentiation [29, 30, 59]. For example, it has been reported that differentiation of HL-60 cells using all-trans retinoic acid resulted in an increase in TOP2B protein [66]. TOP2B protein expression was also up-regulated when NB4 and NB4-MR2 cells were treated with PMA for 2 h, and that an increase in protein expression correlated with an increase in PMA concentration [67]. This study also postulated that this up-regulation could be the result of an up-regulation of protein kinase delta (PKCD), which is stimulated by PMA, and suggests that PKCD phosphorylates TOP2B protein leading to an increase in stability and a decreased rate of degradation. Inhibition of PKCD was also shown to result in a decrease in TOP2B protein level [67]. This could account for why there is a decrease in *TOP2B* mRNA and yet no significant change in the level of TOP2B protein when U937 cells are exposed to PMA for 72 h. However, in an earlier study using HL-60 cells, levels of TOP2B were shown to be reduced upon differentiation with DMSO [68]. The difference in TOP2B levels observed between the studies may be due to the differentiating agent used as well as cell type and the timeframe examined. For example, TOP2B has been shown to be required for late but not early neuronal development and that changes in the level of TOP2B could change over the process of differentiation [25].

Relative levels of *TOP2A* and *TOP2B* mRNA were also examined in primary monocytes differentiated *ex-vivo* using M-CSF. M-CSF acts by indirectly activating Protein Kinase C (PKC) by inducing the production of diacylglycerol (DAG) [69, 70]. The activation of PKC causes a cascade of events leading to activation of transcription factors, and therefore transcription of genes involved in differentiation. This pathway of activation is very similar to that of PMA as PMA is analogous to DAG [71], thus being able to stimulate PKC directly. *TOP2A* mRNA could be detected in only half of the non M-CSF samples examined. This is not unexpected as previously only very low levels of *TOP2A* mRNA levels were detected in peripheral blood mononuclear cells from healthy volunteers with levels equating to only ~ 2.5% of the *TOP2B* mRNA levels seen in the same cells [72]. As well as the known variability of cells between donors [73], CD14 + monocytes do not proliferate [74], thus *TOP2A* expression level is expected to be low per se [75, 76]. No *TOP2A* mRNA was seen in all of the samples cells exposed to M-CSF, except one. This suggests that differentiation into the macrophage-like phenotype, generally results in a reduction of *TOP2A* mRNA. A down regulation of *TOP2A* mRNA was also demonstrated when primary monocytes underwent differentiation with M-CSF using transcriptome analysis [77]. In contrast *TOP2B* mRNA was detected in all of the non M-CSF samples examined. Similarly *TOP2B* mRNA was detectable in peripheral blood cells from all health volunteers and its level varied threefold between volunteers [72]. In all of the four samples treated with M-CSF, levels of *TOP2B* mRNA reduced. In 50% of the samples, *TOP2B* mRNA dropped to less than half of that detected in untreated cells and with *TOP2B* mRNA becoming undetectable in the other 50%. These results are similar to those seen in the cell lines used in the current study (Fig. 2). It would be useful to measure TOP2A and TOP2B protein levels in these cells to determine if the mRNA levels correlate to relative level of proteins. Interestingly several studies have shown a decline in levels of TOP2B protein as cells enter G0, although in contrast to TOP2A, TOP2B protein remains detectable at this time [63, 78, 79].

From our results we suggest that *IL1B* transcriptional silencing seen with epirubicin is not due to the inhibition of TOP2 enzymatic activity. Nevertheless, ICRF-193 does attenuate secretion of bioactive IL-1β. How ICRF-193 does this is yet unknown, but one hypothesis may be that it inhibits stimulus-induced IL-1β post-translational processing. This could be *via* a variety of mechanisms, for example by inhibiting the NLRP3 inflammasome either by direct interaction [80] or by down-regulating the NLRP3 inflammasome components and reducing the release of cleaved caspase-1 *via* modulation of chromatin structure and function [8–13]. It is also possible that ICRF-193 may promote ubiquitination and degradation of inflammatory caspases. Recently, the small molecule kaempferol and a bacterial effector protein have both been observed to do just this and attenuate IL-1β secretion [81, 82] Further studies are needed to examine if ICRF-193 promotes ubiquitination and restraint of the inflammasome and also if it’s effect can be potentiated by using it in combination with other such drugs. In addition it is possible that such effects of ICRF-193 may be visible at earlier time points than those measured in this study. Interestingly, ICRF-193 has also been shown to cause degradation of TOP2B protein *via* the proteasome but not TOP2A [64, 65]. However, these studies have only explored the effect of ICRF-193 up to a maximum of 12 h and at much higher doses. It is also possible that ICRF-193 can alter indirect components which inhibit the inflammasome. For example, Doxorubicin, another anthracycline TOP2 poison, increased expression levels of the dopamine receptor DRD1, which in the presence of an agonist decreased the activation of the inflammasome, attenuating release of IL-1β [83].

In summary, the results provide a novel finding that inhibition of TOP2 activity by ICRF-193 can attenuate the secretion of IL-1β, which warrants further investigation of the mechanism and whether this drug could be further investigated for the treatment of inflammatory disorders. Recently, more water soluble prodrugs of ICRF-193 have been synthesised and show promise pharmaceutically as they display low cytotoxicity and favourable ICRF-193 release [84, 85]. Their ease of synthesis is also important as it represents a cheaper alternative to other recombinant antagonists [86].

## Data Availability

Data is available upon request.
